# Predictors of Thyroid Gland Invasion in Laryngeal Squamous Cell Carcinoma

**Published:** 2018-05

**Authors:** Keyvan Aghazadeh, Sasan Dabiri Satri, Amirsina Sharifi, Maryam Lotfi, Bita Maraghehpour, Arsalan Hashemiaghdam

**Affiliations:** 1 *Otorhinolaryngology Research Center, Tehran University of Medical Sciences, Tehran, Iran. *; 2 *Department of Pathology, Amiralam Hospital, Tehran University of Medical Sciences, Tehran, Iran.*; 3 *International Campus, School of Dentistry, Tehran University of Medical Sciences, Tehran, Iran.*; 4 *Faculty of Medicine, Tehran University of Medical Sciences, Tehran, Iran.*

**Keywords:** Larynx, Neoplasm invasion, Squamous cell carcinoma, Thyroid

## Abstract

**Introduction:**

Laryngeal squamous cell carcinoma (SCC) can invade the thyroid gland leading to unnecessary thyroidectomies with subsequent hypothyroidism and hyperparathyroidism. Thus, clinicopathological variables should be defined in order to predict thyroid gland invasion preoperatively.

**Materials and Methods:**

We performed a retrospective analysis of 1,465 patients with laryngeal SCC referred to our center between March 2009 and January 2016. Among these patients, 60 individuals underwent total laryngectomy and either thyroid lobectomy and isthmectomy or total thyroidectomy.

**Results:**

Thyroid gland invasion was observed in 20% of samples. The following variables were associated with thyroid gland invasion: transglottic spread of the tumor (odds ratio [OR]: 2.04, 95% confidence interval [CI]: 1.15–5.81, P=0.004), thyroid cartilage involvement (OR: 1.53, 95% CI: 0.94–2.50, P=0.02), and anterior commissure involvement (OR: 5.75, 95% CI: 0.86–38.42, P=0.01). In addition, the largest dimension of the tumor was significantly associated with thyroid gland involvement (r=0.36, 95% CI 0.05–0.67, P=0.004). Multivariate linear regression analysis confirmed these findings.

**Conclusion:**

The rate of thyroidectomies performed in cases of laryngeal SCC is much higher than the actual rate of thyroid gland invasion. Thus, preoperative evaluation to find transglottic spread of the tumor, thyroid cartilage, and anterior commissure involvement should be considered.

## Introduction

Despite the low prevalence of thyroid gland involvement in laryngeal squamous cell carcinoma (SCC), the selection of appropriate patients for thyroidectomy has proved problematic (1,2). Previous reports have indicated that 1–30% of advanced laryngeal SCC invades the thyroid gland (3-5). 

This invasion mainly occurs through direct extension as the thyroid and larynx have an anatomical proximity. Gaillardin et al. stated that approximately 75% of patients with advanced laryngeal SCC undergo unnecessary thyroidectomy (6), causing hypothyroidism and hypoparathyroidism in 23–63% and 25–52% of cases, respectively (7,8). During recent decades, there has been a shift towards more preservative surgical approaches, and total laryngectomy has become the final option in advanced cases or as salvage therapy following the failure of noninvasive treatments (9). Although the American Society of Clinical Oncology abandoned the larynx preservation approach in patients with thyroid gland invasion (10), later studies showed chemo-radiation therapy to be able to control the disease as if an organ preservation approach had been followed (11,12). Thus, there is a need for clinical and histopathological variables to guide the treatment approach and the surgical extent of thyroid resection. This approach ensures that treatment goals are achieved and major complications are avoided (13,14).

In the present article, our primary purpose was to calculate the frequency of thyroid gland involvement in patients with advanced laryngeal SCC undergoing total laryngectomy and concomitant lobectomy and isthmectomy or total thyroidectomy. The second objective was to determine if certain clinical and histopathological characteristics predict thyroid gland invasion.

## Materials and Methods

We performed a retrospective review of all consecutive patients undergoing total laryngectomy for advanced laryngeal SCC at Amir Alam Hospital, Tehran University of Medical Sciences, Tehran, Iran from March 2009 to January 2016. The institutional review board and the ethics committee of Amir Alam Hospital approved the study protocol. Patients were enrolled as if they had undergone total laryngectomy and either thyroid lobectomy and isthmectomy or total thyroidectomy. Patients with evidence of distant metastatic disease, second primary tumors, histologic findings other than SCC, or radiotherapy or chemotherapy prior to surgery were excluded. The actual number of patients who suffered from laryngeal SCC during the study period was 1,465 individuals, but only 60 were eligible to enter the study.

Thyroidectomy was considered whenever subglottic extension or direct thyroid gland involvement was detected. Ipsilateral thyroidectomy was performed in all lateralized cancers. In addition, patients with bilateral lesions or with palpable or firm contralateral nodules, clinically evident calcification, or the presence of a clinically apparent node on examination underwent total thyroidectomy. Histopathological analysis of all specimens was performed and reviewed retrospectively. Thyroid gland invasion was addressed according to histopathological reports. The American Joint Committee on Cancer (AJCC) Cancer Staging Manual, 7th Edition, 2010 was applied to tumor, node, metastasis (TNM) staging of all patients.Upper aerodigestive endoscopic biopsies and contrast-enhanced neck computerized tomography was performed as pre-surgical assessments in all patients. All surgical procedures were performed by a single experienced team.

 The following characteristics were analyzed: demographics, smoking habit, tumor features such as stage, grade, site, size, thyroid gland histopathology, thyroid cartilage involvement, anterior commissure involvement, and perineural and lymphovascular invasion.

Qualitative variables are presented as frequency and were analyzed using the two-tailed Fisher’s exact test. Quantitative variables are presented as a mean ± standard deviation (SD) and were analyzed using an analysis of variance (ANOVA) test. The odds ratio (OR) and its 95% confidence interval (CI) was applied to assess the degree of association between thyroid gland tumoral involvement and other variables. 

All analyses were performed with a two-sided method using Statistical Package of Social Science software (SPSS) version 22 (SPSS, Inc., Chicago, IL), and a P<0.05 was defined as statistically signiﬁcant.

## Results

Of 60 patients, 57 (95%) were male, and three (5%) were female. The mean patient age (±SD) was 60.98 ±8.83 years, ranging from 40 to 80 years. The majority of patients (93.3%) had a previous or current history of smoking. The tumor was located at the transglottic, supraglottic, and glottic site in 45, eight, and six cases, respectively. Stage T3 laryngeal SCC was found in 20 (33.3%) cases, and 40 patients (66.7%) suffered from stage T4 carcinoma. The tumor was well differentiated in 36 (60%) patients and was moderately differentiated in 24 (40%) patients. The largest dimension of the tumor was 5.28±1.69 mm.

Patients with and without thyroid involvement were compared, and the following characteristics were found to be significantly different between two groups: stage (P=0.04), grade (P=0.01), mean of largest tumor dimension (P<0.0001), site of laryngeal tumor (P=0.02) (Table.1).

**Table 1:** Comparison of clinicopathological data between patients with and without thyroid involvement

**Characteristic**		**Thyroid gland invasion**	**No thyroid gland invasion**	**P-value**
Age (mean±SD)		61.00±9.69	60.97±8.71	0.99
Gender (n,%)	Male	10 (83.3%)	43 (89.5%)	0.61
	Female	2 (16.7%)	5 (10.5%)	
T subtype (n,%)	T3	11 (91.7%)	29 (60.5%)	0.04
	T4	1 (8.3%)	19 (39.5%)	
Grade (n,%)	Well differentiated	11 (91.7%)	25 (52%)	0.01
	Moderately well differentiated	1 (8.3%)	23 (48%)	
Largest dimension of the tumor (mean±SD)		6.50±1.69	4.96±1.36	0.004
Site of laryngeal tumor (n, %)	Transglottic	6 (50%)	40 (83.4%)	0.008
	Supraglottic	4 (33.4%)	4 (8.3%)	
	Glottic	2 (16.6%)	4 (8.3%)	
Thyroid surgery	TT	4 (33.4%)	20 (41.5%)	0.86
	L	7 (58.3%)	23 (48%)	
	PL	1 (8.3%)	5 (10.5%)	

TT: Total thyroidectomy, L: Lobectomy, PL: Partial Lobectomy

**Table 2:** Comparison of tumoral pathology data between patients with and without thyroid involvement

**Characteristic**		**Thyroid gland invasion**	**No thyroid gland invasion**	**P-value**
Lymph node invasion	Positive	6 (50%)	15 (31.2%)	0.31
	Negative	6 (50%)	33 (68.8%)	
Vascular invasion	Positive	1 (8.3%)	12 (25%)	0.43
	Negative	11 (91.7%)	36 (75%)	
Perineural invasion	Positive	2 (16.6%)	5 (10.4%)	0.61
	Negative	10 (83.4%)	43 (89.6%)	
Thyroid cartilage involvement	Positive	7 (58.4%)	43 (89.6%)	0.02
	Negative	5 (41.6%)	5 (10.4%)	
Anterior Commissure involvement	Positive	11 (91.7%)	25 (52%)	0.01
	Negative	1 (8.3%)	23 (48%)	

Table 2 presents the histopathological properties of each cluster. For categorical variables, we used OR to assess the association between exposure and outcome. In this regard, stage T4 (OR: 4.75, 95% CI: 0.70–32.03, P=0.04), moderate differentiation (OR: 5.75, 95% CI: 0.86–38.42, P=0.01), transglottic spread of the tumor (OR: 2.04, 95% CI: 1.15–5.81, P=0.004), thyroid cartilage involvement (OR: 1.53, 95% CI: 0.94–2.50, P=0.02), and anterior commissure involvement (OR: 5.75, 95% CI: 0.86–38.42, P=0.01) were associated with thyroid gland involvement. For nominal variables, Spearman's correlation coefficient test revealed that the largest dimension of the tumor is significantly associated with thyroid gland involvement (r=0.36, 95% CI: 0.05–0.67, P=0.004).

Multivariate linear regression analysis (forward method) was used to test whether the observed association is confounded by age, gender, lymph node invasion, vascular invasion, or perineural invasion. Outcome in this analysis was thyroid gland invasion, defined according to whether or not the thyroid gland was invaded by tumoral cells. It was revealed that stage T4, moderate differentiation, the transglottic spread of the tumor, thyroid cartilage involvement, and anterior commissure involvement were all independent variables predicting thyroid gland invasion.

## Discussion

In this study we reported that thyroid gland invasion was found in 20% of patients undergoing thyroidectomy of any extent. This is in line with previously reported values ranging from 1% to 30% (15). Although it has been proven that thyroid involvement is linked with poor prognosis (1,8), our series showed that the thyroidectomy pathology report was suggestive of no tumoral involvement in 80% of cases. Thus, the risk of catastrophic complications such as hypothyroidism and hyperparathyroidism makes it necessary to reevaluate indications for thyroidectomy (7, 16). Mangussi-Gomes et al. (14) reported a series of 83 patients with T3 and T4 laryngeal SCC who underwent concomitant total laryngectomy and thyroidectomy. In this series, there was no significant relationship between lymph node staging, angiolymphatic invasion, and perineural invasion with thyroid gland involvement. Also, in our study, the association of lymph node staging, angiolymphatic invasion, and perineural invasion did not reach statistical significance. This finding confirms the idea that laryngeal SCC invades the thyroid gland mainly through direct invasion (13).

There is a general perception that less differentiated and larger tumors are more likely to invade into adjacent structures. In our study, we showed that moderately differentiated tumors which are graded II and tumors with larger dimensions have a greater risk of invading the thyroid gland. Evidence suggests that less differentiated tumors do not necessarily behave aggressively (2, 14). However, transglottic spread, which has been shown by many authors to be associated with thyroid gland invasion (3, 4, 13, 17), means that a lesion was large enough to invade both sides of the glottic region.

It was revealed that transglottic spread of the tumor, anterior commissure or thyroid cartilage involvement are positively associated with positive thyroid glands for carcinoma. Previous studies have demonstrated a similar association (6, 18). The same logic about the anatomical proximity of the thyroid gland and larynx and the fact that there is only a weak laryngeal framework, including the cricothyroid membrane and the paramedian cricothyroid space, between the thyroid gland and the larynx might explain these data. In 2009, a meta-analysis study was performed using eight series, and proposed that thyroidectomy is indicated whenever transglottic tumors, subglottic tumors, and tumors with subglottic extensions >10 mm are present (13). Previous authors had tried to introduce selection criteria to perform concomitant thyroidectomy. In many cases, it included anterior commissure involvement, subglottic space invasion, and stage T3–T4 transglottic spread of laryngeal cancer (3,17,19). Cartilage ossification occurs when new vascularization appears as part of a normal aging process (20), and it has been hypothesized that tumor cells can use new vessels to invade (21). Indeed, Gallo et al. and Gregor et al. suggested laryngeal SCC was capable of activating osteoblasts and osteoclasts to accelerate cartilage ossification (22,23).

The decision to perform thyroidectomy and its extension should be based on a preoperative assessment of the tumoral involvement of the structures related to the thyroid gland. In this regard, magnetic resonance imaging has been introduced to correctly predict thyroid gland invasion, with a sensitivity and specificity of 100% and 97.4%, respectively (22).

This study has inherent limitations and strengths. The retrospective cross-sectional nature of the design prevents us from relying on the results entirely, and highlights the need for clinicopathological factors to be investigated through randomized trials to accurately define indications for thyroidectomy and its extent in the context of laryngeal SCC. Although we performed this study in a tertiary center with 1,465 patients with laryngeal SCC throughout the study period, the fact that we selected those patients who had undergone thyroidectomy diminished the impact of the large sample size; although it was not possible to assess thyroid gland histopathology in all patients.

## Conclusion

The rate of thyroidectomies performed in cases of laryngeal SCC is much higher than the actual rate of thyroid gland invasion. Thus, this procedure should be confined to cases with preoperative evidence of anterior commissure or thyroid cartilage involvement and transglottic spread of the tumor.
